# External proficiency testing for histocompatibility and immunogenetics in today and future

**DOI:** 10.3389/fgene.2024.1294330

**Published:** 2024-02-26

**Authors:** Fatma Savran Oguz

**Affiliations:** Tissue Typing Laboratory, Department of Medical Biology, Istanbul Faculty of Medicine, Istanbul University, Istanbul, Turkiye

**Keywords:** external proficiency testing, EPT, HLA, quality control, QA

## Abstract

The Histocompatibility and Immunogenetics laboratories provide disease association and pharmacogenetic analyses as well as the tests required for transplantation immunology and transfusion medicine. They perform Human Leukocyte Antigen (HLA) genotyping in patients/recipients and potential donor candidates for solid organ and stem cell transplants using various molecular methods, and determine mismatches. In addition, they also perform HLA antibody tests to detect anti-HLA antibodies in patients and flow cross-matches to evaluate donor-recipient compatibility. Evidence-based clinical guidelines have emphasized the importance of laboratory tests in clinical practices for a long time. Understanding the principles of Quality Control and External Quality Assurance is a fundamental requirement for the effective management of Tissue Typing laboratories. When these processes are effectively implemented, errors in routine assays for transplantation are reduced and quality is improved. In this review, the importance of Quality Assurance, Quality control and proficiency testing in Histocompatibility and Immunogenetic testing, the necessity of external proficiency testing (EPT) for accreditation, and existing and potential EPT programmes will be reviewed and evaluated in the light of the literature.

## Introduction

Histocompatibility and Immunogenetics Laboratories (Tissue Typing Labs) play an active role in both solid organ and hematopoetic stem cell transplants. Histocompatibility testing is essential for donor identification and risk assessment in solid organ and hematopoietic stem cell transplant. Additionally, it is useful for identifying donor specific alleles for monitoring donor specific antibodies in post-transplant patients.

Post-transplant chimerism test to evaluate engraftment especially in hematopoietic stem cell transplantation (HSCT) transplant patients and donor specific antibody monitoring in renal transplants are routine tasks. In addition, Human Leukocyte Antigen (HLA) Laboratories provide disease association and pharmacogenetic analyses as well as the tests for transfusion medicine.

In recent surveys performed with expert clinicians in Germany and United States, it was reported that 60%–70% of clinical decisions were influenced by the results of laboratory tests performed both in hospitals and in external centers (Rohr et al., 2016). Evidence based clinical guidelines indicate that at least 80% of guidelines, targeting to make a diagnosis or manage a disease, require laboratory tests (Goodman et al., 2005). Laboratories have been aware of this for a long time and try to reduce the risk of misinterpretation of the test results obtained from different laboratories (Shahangian and Cohn, 2000) However, these concepts should be based on well-designed and well-implemented Quality Control (QC) and External Quality Assurance (EQA) systems (Badrick, 2003; Badrick, 2021).

**FIGURE 1:**
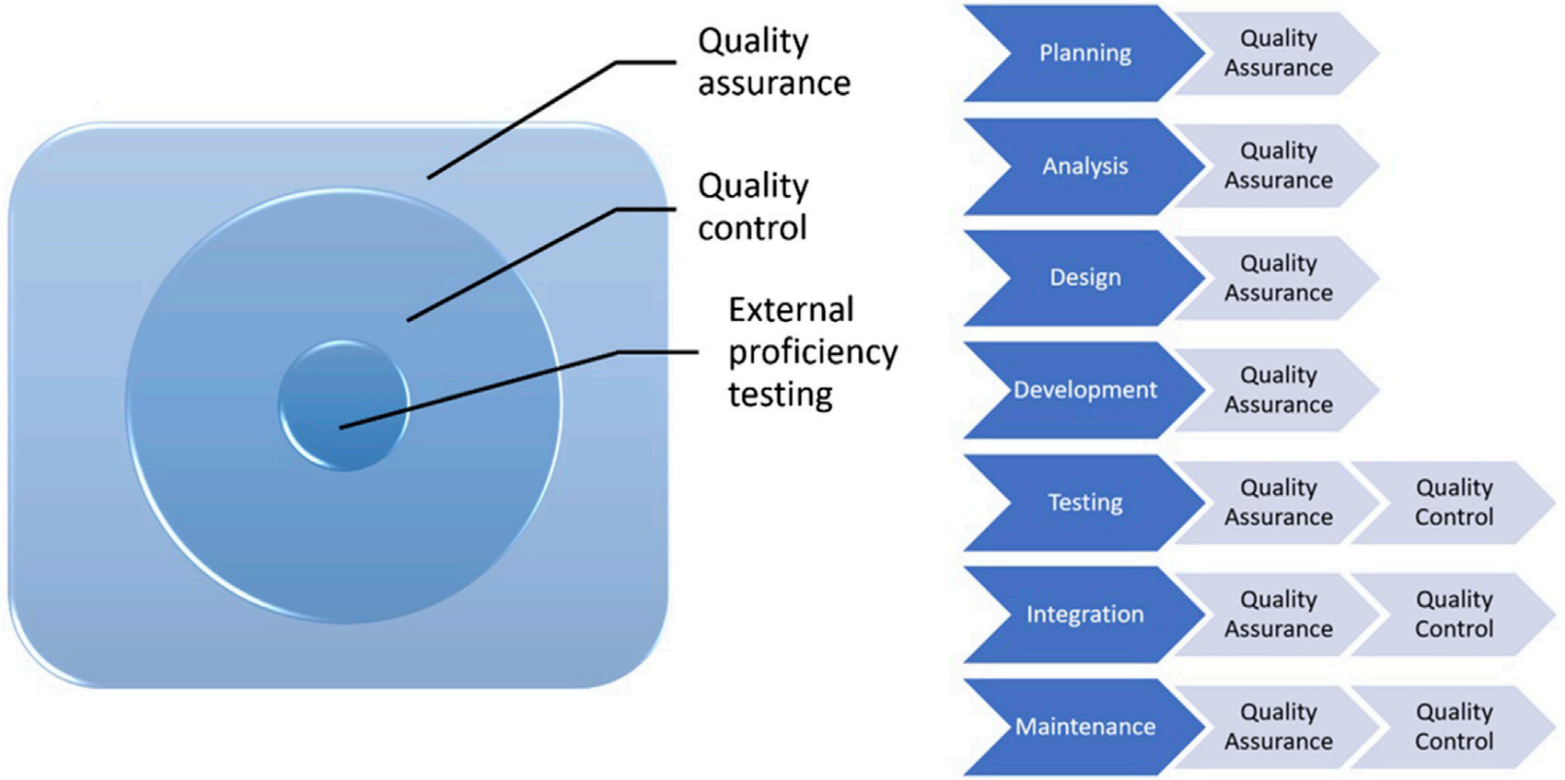
Processes from QA to EPT.

Quality assurance (QA) is a subgroup of quality management (Figure 1). It is proactive, concerns the whole process, includes a series of activities and procedures, that occur during operations and help in providing a high quality analysis, and prevents errors. All health institutions should establish QA policies for laboratories to meet these standards for each analysis. It should be kept in mind that quality control is a part of quality assurance.

QC involves establishment of a quality standard or specifications for each aspect of a test procedure, specification of how the test procedure complies with quality standards and taking the necessary corrective measures to bring up the procedures to standard. It is an active and team-wide process that identifies the errors related to all outputs during the procedure and after the procedure.

Internal Quality Control tests have been designed to control if a test or procedure will produce the same result in case of in-laboratory variations or when performed by varying technicians.

External Quality Control (EQC) is defined as an evaluation study performed by an exteral provider using samples with known or unknown content or concentration with the objective of providing or improving the reliability of laboratory test results. EQC programmes are conducted by independent institutions and comparatively evaluate the performances of test results and reports of laboratories. With EQC programmes, laboratories’ performances are compared with the performances of other laboratories and evaluated on an international scale.

Demonstrating compliance of Tissue Typing laboratories with good practices in providing clinical transplantation services has gained importance, and there are many legal and regulatory requirements targeting to provide appropriate review and documentation of services. Therefore, laboratories should provide assurance related to the quality of the services they give in line with regulatory objectives (Harmer et al., 2018).

The increase in the number of laboratories and methods in years proved the necessity of meeting a high standard for the results reported by different laboratories. In this context, standardization studies and survey programmes were established many years ago.

In a study which summarized the studies, in which HLA standardization by way of international cell exchange was performed, tissue typing performed using a blind design in 468 people in a 12-year period was examined. The number of participant laboratories was reported to be increased from 85 in the beginning to 285. It has been emphasized that these standardization studies help to standardize typing reactives of tissue typing laboratories globally, to determine new specificities and to evaluate the status of improvement in tissue typing in renal transplant practices (Loon et al., 1987) The Histocompatibility Survey Programmes was organized in 1982 by American Society for Histocompatibility and Immunogenetics (ASHI) and College of American Pathologists (CAP) as a joint project to evaluate laboratory performance in HLA typing, lymphotoxicity crossmatch and antibody analysis (Marrari and Duquesnoy, 1994).

The risk of post-transplant complications is reduced by way of detailed analysis of the patient’s anti HLA profile and appropriate donor-recipient matching. Precise characterization of alloantibodies in sensitized patients and complete HLA typing at the allelic level are mandatory at the time of transplantation (Morath et al., 2012). Furthermore, knowledge of HLA sensitivities and identification of anti-HLA antibodies among potential kidney recipients is essential to control graft loss (Duquesnoy et al., 2016).

Appropriate techniques should be used to increase the reliability of the tests performed for histocompatibility which is accepted to be effective in graft loss, and a meticulous quality control system should be implemented. With this objective, various EPT programmes were established globally (Table 1). Successful performance in EPT was accepted as a prerequisite for accreditation of a laboratory (Doxiadis et al., 2000; Bogunia-Kubik et al., 2006; Bogunia-Kubik and Lange, 2008; Bogunia-Kubik and Lange, 2010; Balza et al., 2023; European Federation for Immunogenetics, 2023).

**TABLE 1:** EPT provider and EFI regions (based on data from European Federation of Immunogenetics, 2023).

EFI region	Regions	EPT
Region 2	Blux	• Eurotransplant (ET), Leiden
		• High Resolution EPT, Maastricht
Region 3	United Kingdom and Ireland	• United Kingdom NEQAS for H&I, Pontyclun
		• United Kingdom NEQAS for Leucocyte Immunophenotyping, Sheffield
Region 4	Germany	• INSTAND e.V., Düsseldorf
		• DZA, Munich
Region 5	Central Europe	• CET, Vienna
		• HLA Proficiency Testing for Central and East Europe, Wroclaw
		• HLA, Prague
Regions 6 + 11	France + Switzerland	• LNRH, Geneva
		• SFHI, Hôpital Saint-Louis, APHP, Paris
Region 7	Italy	• IT EPT, Rome
		• EPT Milan
Region 8	Balkans + Israel	• Sofia (Balkan External Proficiency Testing FCXM, CDCXM, PRA)
		• Istanbul (Balkan External Proficiency Testing FCXM, CDCXM, HLA)
Regions 9 + 10	Iberia	• GECLID-SEI, Valladolid
Other Regions	outside Europe	• UCLA International DNA Exchange, Los Angeles

EFI, european federation for immunogenetics; EPT, external proficiency testing; HLA, human leukocyte antigen; FCXM, flow cytometry crossmatch; CDCXM, Complement-dependent Microcytotoxicity Crossmatch; PRA, panel reactive antibody.

**FIGURE 2:**
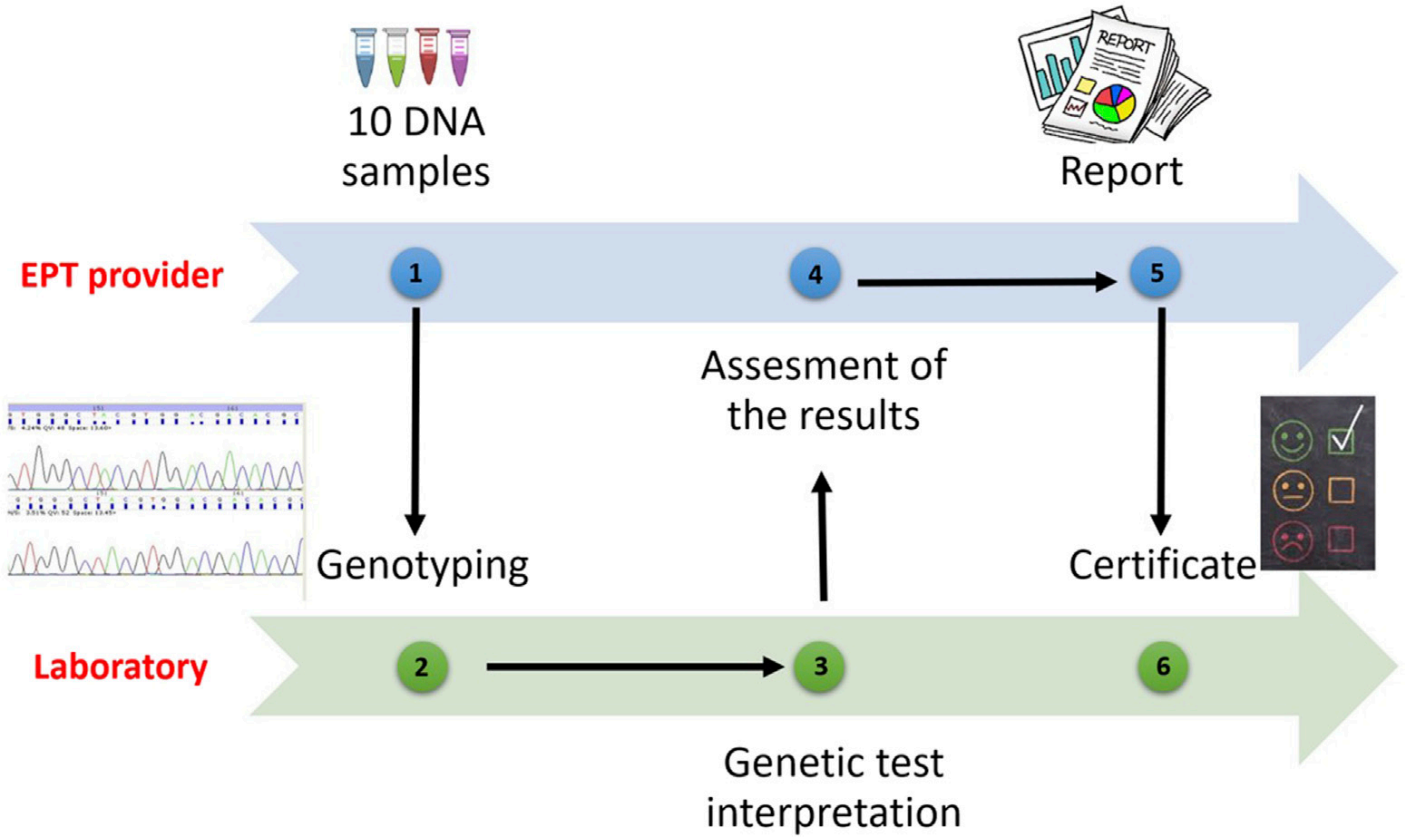
EPT Programmes process.

All laboratories applying to receive accreditation from the European Federation for Immunogenetics (EFI) or wishing to resume their accreditations, are obliged to participate in EPT programs related to laboratory practices involving the categories for which they will be accredited (HLA typing, antibody screening and detection, cross match, etc.) (European Federation for Immunogenetics, 2023) (Figure 2).

If there are no programmes specified for a certain category, the laboratory should participate in an EPT workshop or trial provided by an EPT provider or be involded in an inter-laboratory sample exchange programmes. The laboratory shall have a predetermined policy for testing EPT specimens, documenting relevant EPT programmes or workshops prospectively on an annual basis. Thus, participation in external proficiency testing workshops will give the opportunity to validate HLA typing results. It will contribute to the training of laboratories by making comparisons with other participants. It is also expected that the error rates of participating laboratories will decrease over the years.

According to the EFI standards, EPT samples should be tested and interpreted one by one or in association using the techniques used routinely for clinical samples. If the same sample is being tested for multiple accreditation categories, the results should be analyzed independent from each other. The annual minimum sample number for EPT is shown in the table (Table 2). If the same sample is being tested with multiple techniques in the same accreditation category, the laboratory should give the provider only one report, but keep the results obtained with different techniques ready for inspection (European Federation for Immunogenetics, 2023).

**TABLE 2:** EPT methods and samples (based on data from European Federation of Immunogenetics, 2023).

Methods	Minimum number of samples for EPT per year
Serological typing	10 samples
Each low resolution DNA-based typing technique	10 samples
Each high resolution DNA-based typing technique	10 samples
Each allelic resolution DNA-based typing technique	10 samples
HPA/HNA/KIR/MICA typing	10 samples
HLA antibody detection	10 samples for HLA class I and 10 samples for HLA class II
	The same samples can be used for the detection of both classes
HLA antibody identification by CDC	10 samples
HLA antibody identification by solid phase assays	10 samples
HPA/MICA antibody detection and identification	5 samples
Crossmatching	20 tests of different donor/recipient combinations of each accredited cell
Haematopoietic chimaerism and engraftment monitoring	10 tests of different donor/recipient mixtures in the range 0%–100% excluding the reference donor and recipient samples

EPT, external proficiency testing; HLA, human leukocyte antigen; HPA, human platelet antigen; HNA, human neutrophil antigens; KIR, Killer Cell Immunoglobulin-Like Receptors; MICA, Major histocompatibility complex class I chain-related genes A; CDC, Complement-dependent Microcytotoxicity.

In recent years, experimental transplant models have shown that mechanisms other than T-lymphocyte anti-donor responses could be effective (Oberbarnscheidt et al., 2014; Dai et al., 2017). In addition, a few translational genetic association studies have showed that incompatibilities originating from interaction of two different genomes could lead to complex immune responses in solid organ transplants and non-HLA antibodies could also be effective in rejection (Sankaran et al., 1999; Grinyó et al., 2008; Menon et al., 2015; Zhang et al., 2021; Jethwani et al., 2022).

Numerous studies in solid organ transplantation provide evidence that high levels of donor-derived cell-free DNA (DD-cfDNA) correlate with clinically relevant endpoints. Increased DD-cfDNA has been associated with episodes of graft injury and rejection. Efforts are ongoing to further improve sensitivity and specificity. DD-cfDNA could be used as a biomarker in the near future as it is quantitative and has the potential to be cost-effective. Although there are EPT programs on cell-free DNA in different fields, EPT programs in transplantation are not yet available (Samoila et al., 2020; Edwards et al., 2022)

Multi-center studies have shown that specification of non-HLA loci with long-term allograft results and identification of non-HLA antibodies in patients might enable sensitive matching of organs in patients who have multiple potential donors. Performance of routine tests in HLA laboratories addressing these parameters will undoubtedly contribute to successful organ transplantation to a great extent. When EPT programmes are examined, it is observed that there is currently no study dedicated to these analyses.

## Conclusion

The role of the Histocompatibility and Immunogenetic laboratories in stem cell and organ transplants has expanded to provide HLA antibody detection and tracking for selection of compatible donors and monitoring desensitization therapies.

In the future, they will request new programmes in accordance with clinical needs in order to perform new routine tests successfully in parallel with accreditation categories and to evaluate and improve laboratory performance.
